# Female mating status has been overlooked in mate choice research: a comment on Richardson and Zuk

**DOI:** 10.1093/beheco/arac099

**Published:** 2023-03-28

**Authors:** Varpu Pärssinen, Charlotta Kvarnemo

**Affiliations:** Department of Biological and Environmental Sciences, University of Gothenburg, Box 463, SE-40530, Gothenburg, Sweden; Department of Biological and Environmental Sciences, University of Gothenburg, Box 463, SE-40530, Gothenburg, Sweden

The study of mate choice and sex roles has come a long way from the assumption that only males are promiscuous and females are coy. However, fragments of this mindset might still influence our research today. In their review, Richardson and Zuk (2022) bring forward the important issue that many mate choice experiments use only virgin females for measuring female preferences, despite the fact that in systems with multiply mating females, most of the mating events should occur with non-virgin females. As full monandry has been found to be much rarer than polyandry in most animal species (Taylor et al. 2014), this is an issue that most mate choice studies should consider carefully.

We find Richardson and Zuk’s review both informative and eye-opening. This is so, despite the fact that their meta-analysis did not reveal large biases in mating preferences between virgin and non-virgin females in previously published literature. Their review still shows how using virgin females is the standard of most mate choice studies, and that only a few papers explicitly state that mated females were used. We strongly agree that more studies should be aware of the potential shortcomings that arise from this discrepancy between experimental animals and wild animal populations.

This problem has gone largely unnoticed until now, as even in the otherwise comprehensive book on mate choice by Rosenthal (2017), virgin females are only mentioned when discussing potential male preferences. While the meta-analysis by Richardson and Zuk did not find differences in choosiness between virgin and mated females, this might be due partly to the low number of publications with non-virgin females and thus warrants further investigating. More studies comparing experimentally these differences within single systems would be especially helpful to evaluate if this is a large concern for mate choice research.

There are several reasons why using virgin females might be justified in mate choice studies. Virgin females are often seen as a “clean slate” unbiased by previous mating experience. A study on reproductive isolation might therefore be more interested in investigating innate female preferences, and using virgin females may yield more reliable results through this approach. However, when estimating the overall reproductive isolation between populations or species, the role of behavioral isolation in restricting gene flow might be seriously underestimated if learned mate recognition is not taken into account (Magurran and Ramnarine 2004). We therefore agree with the Richardson and Zuk in that future studies should consider whether the mating status of their females fully represents the natural conditions.

Richardson and Zuk choose to limit their focus to studies of three categories: reproductive isolation, inbreeding avoidance and sexually transmitted disease. We are impressed with the thoroughness of the meta-analysis in this framework, but also recognize that it leaves out some large categories of mate choice research. For example, we still lack corresponding knowledge of studies that measure sexual selection acting on male phenotypic traits. If the use of virgins is widespread in this field as well, we could expect potential underestimation of female choosiness. The authors also do not consider the effects of different mating systems in their analysis, but it might warrant further study. In systems where females are significantly mate-limited, virgin females may have a stronger reason to be less choosy than mated females. The differences in choosiness between virgin and mated females might therefore be the largest in species where females also compete for access to males (Hare and Simmons 2019).

The authors caution future researchers to be aware of the unknown mating status of wild-caught females. However, we would argue that a sufficiently large sample of wild-caught females should be the best representation of the types of potential mates the males would encounter in the wild. Using only wild-caught females would therefore avoid most of the concerns presented by this review, at least when it comes to estimating realistic female preferences in wild populations.

In summary, Richardson and Zuk raise an important concern for mate choice studies. Future research needs to take this issue into careful consideration. In addition, we think that the extent to which mating status might affect choosiness deserves to be delved into in its own right, and in both sexes, to estimate behavioral differences between virgin and non-virgin individuals.
